# A guide to modern statistical analysis of immunological data

**DOI:** 10.1186/1471-2172-8-27

**Published:** 2007-10-26

**Authors:** Bernd Genser, Philip J Cooper, Maria Yazdanbakhsh, Mauricio L Barreto, Laura C Rodrigues

**Affiliations:** 1 Department of Epidemiology and Population Health, London School of Hygiene and Tropical Medicine, London, UK; 2 Instituto de Saúde Coletiva, Federal University of Bahia, Salvador, Brazil; 3 Centre for Infection, St George's University of London, London, UK; 4 Instituto de Microbiologia, Universidad San Francisco de Quito, Quito, Ecuador; 5 Department of Parasitology, Leiden University Medical Center, Leiden, The Netherlands

## Abstract

**Background:**

The number of subjects that can be recruited in immunological studies and the number of immunological parameters that can be measured has increased rapidly over the past decade and is likely to continue to expand. Large and complex immunological datasets can now be used to investigate complex scientific questions, but to make the most of the potential in such data and to get the right answers sophisticated statistical approaches are necessary. Such approaches are used in many other scientific disciplines, but immunological studies on the whole still use simple statistical techniques for data analysis.

**Results:**

The paper provides an overview of the range of statistical methods that can be used to answer different immunological study questions. We discuss specific aspects of immunological studies and give examples of typical scientific questions related to immunological data. We review classical bivariate and multivariate statistical techniques (factor analysis, cluster analysis, discriminant analysis) and more advanced methods aimed to explore causal relationships (path analysis/structural equation modelling) and illustrate their application to immunological data. We show the main features of each method, the type of study question they can answer, the type of data they can be applied to, the assumptions required for each method and the software that can be used.

**Conclusion:**

This paper will help the immunologist to choose the correct statistical approach for a particular research question.

## Background

The understanding of the importance of immunological mechanisms underlying human disease and the identification of associated immunological markers have grown enormously over the past ten years and the number of published immunological studies that investigate the relationships between human disease and cytokines and other immunological parameters has increased rapidly. Technical developments in sample processing and sophisticated immunological techniques permit the analysis of more immunological parameters in larger samples of human subjects, containing information that allows not only the measurement of simple associations between two parameters but also the exploration of the complex relationships between immunity, disease, environmental, social and genetic factors. The potential complexity of the possible relationships between large numbers of immunological parameters poses a special challenge for the applied immunologist: how to select the appropriate statistical techniques to extract the maximum relevant information from complex datasets and avoid spurious findings.

Immunologists tend to use simple statistical approaches even when multiple relationships between immunological parameters are expected, [1,2] instead of multivariate statistical approaches that can analyse simultaneously multiple measurements on the same individual. Multivariate statistical analysis techniques are being widely applied in other scientific fields and numerous books and articles have been published that describe these techniques in detail. Unfortunately, this literature is not easily accessible to the applied immunologist without a detailed knowledge of statistics and few articles have been written demonstrating the application of statistical techniques to immunological data [3,4].

This paper provides an overview of statistical analysis techniques that may be considered for the analysis of immunological data. We discuss specific aspects of immunological studies, give examples of typical scientific questions related to immunological data and present a statistical framework to help the immunologist to choose the correct statistical approach for a particular research questions. Although we have focused on cytokine data in the examples provided, the methods presented are applicable to most other immunological parameters.

## Specific aspects of immunological studies relevant for statistical analysis

In the following section we discuss specific aspects of immunological studies that are relevant for statistical analysis.

### Structure of immunological data

Before analysing immunological data it is very important to examine the structure of the data because most statistical methods will only give the correct answer if the data has the characteristics required for the use of that method ("satisfy the data assumptions"). For example, common data assumptions are that the observations are approximately normally distributed or that the variances are similar across different subpopulations. Unfortunately, immunological data very frequently do not meet these assumptions and investigators are obliged to either apply data transformations (e.g. a logarithmic transformation to make skewed data approximately normally distributed), or to choose an alternative statistical techniques with less stringent data assumptions (e.g. using a non-parametric statistical approach that does not require the data to be normally distributed [5] instead of a parametric statistical technique that does). A further important aspect of immunological data is that different immunological parameters measured in the same study subject are frequently highly correlated ("multicollinearity"). Hence the application of statistical techniques that assume independence among the observations is often not valid and in such situations a method should be used that takes into account the fact that study variables may be the result of a common underlying biological mechanism. Examples of underlying biological mechanisms that can not be directly observed but will influence that value of more than one immunological variable are: "immune maturation," "down regulation" or "Th2 shift."

### Complexity of the relationships in immunological parameters

Immunological parameters are often involved in complex immunological mechanisms; and relationships between immunological parameters may be changeable. For example, a specific parameter (e.g. a cytokine) may have different effects in different cell populations, at different times and in the presence (or absence) of other immunological parameters. We often aim to explain the complete causal pathway from a non-immunological factor (e.g. exposure to an allergen) to an outcome (e.g. atopy or asthma). Clearly simple univariate statistical analysis would not be able to identify such inter-relationships among several study variables and the underlying immunological mechanisms that cannot be measured; multivariate statistical techniques are required that can examine multiple parameters simultaneously. A fundamental step to guide statistical analysis is to make the hypothesis explicit in a conceptual framework [6]. *Conceptual frameworks *present the proposed inter-relationships among the study variables and define any larger underlying immunological mechanisms assumed to influence their values. Conceptual frameworks should be detailed and explicit as they are used to guide the analysis.

Two further important aspects of immunological data that are not the focus of this paper but should be mentioned are:

### Reproducibility of the measurement of an immunological parameter

*Reproducibility *reflects how often we obtain the same result using the same laboratory test and sample. Some variation is expected for any measurement and statistical analysis must take into account the degree of variation. Although reproducibility of immunologic measurements is well defined for some immunological outcomes, particularly those used for diagnostic purposes, such as antibody levels associated with vaccine protection (e.g. levels >10 IU/mL for anti-HBS for vaccine response to hepatitis B vaccine), and for phenotypic characterization (CD4 counts or CD4/CD8 ratio for evaluation of immune status in HIV), reproducibility is not well defined for most immunological parameters. This is independent of the separate but important issue of repeatability between centres in multicentre studies that measure the same immunological parameters in different laboratories or even the measurement of the same parameter between different studies.

### Multiple testing

The problem of *multiple testing *is becoming increasingly relevant in immunological studies as the number of immunological parameters that can be measured increases and investigators conduct a large number of statistical tests on the same study data [7]. A specific concern in statistical analysis is to separate associations that occur by chance (because of "random variation" or "noise") from those reflecting true biological relationships ("systematic variation," often assumed to be a causal relationship). Most researchers use a statistical significance level ("type I error," for example P = 0.05) to decide whether the result of an analysis is likely to be due to chance. Conducting multiple hypothesis testing may result in substantial inflation of type I errors (depending on the degree of dependence between the tests). For example, if the value p < 0.05 is used, conducting twenty independent significance tests within a data set is likely to result in one comparison being significant just by chance. There are numerous multiple comparison procedures to adjust statistical analysis for type I error inflation, for example simultaneous test procedures, such as the approaches by Bonferroni, Tukey, Scheffé or Dunnet or more sophisticated step-wise procedures, such as the techniques by Newman-Keuls or Ryan. A good overview about the most important multiple comparison techniques is given by Toothaker [8].

## Research objectives of immunological studies

In this section we list typical research questions from immunological studies.

Common objectives of immunological studies can be grouped into four overall categories:

i) Those that *investigate patterns of associations between several immunological parameters*, without assuming any causal relationship (and therefore not classifying study variables as dependent variables (i.e. outcomes) and independent variables (i.e. explanatory variables or covariates).

For example, typical research questions of such studies are:

• *To assess the magnitude of the correlation between different cytokines *or to quantify the balance between levels of cytokine expression. For example, the research question might be to measure the correlation between pro-inflammatory and anti-inflammatory cytokines (e.g. correlation between TNF-*α *and IL-10) or to quantify the "balance" between pro-inflammatory and anti-inflammatory cytokines (e.g. by calculating the ratio TNF-*α*/IL-10).

• *To identify highly correlated cytokines and to place them into groups *which reflect an unobserved underlying mechanism. For example, Th1-related immune responses such as IFN-*γ *and TNF-*α *may mediate an inflammatory disease. Depending on the question being investigated, it may be more appropriate to first use a statistical analysis approach to "reduce the data", i.e., to aggregate the correlated Th1 related cytokines to form a "summarising variable" that reflects the underlying immunological mechanism (e.g. "degree of Th1 immune response") and use that summary variable in the analysis rather than using all the variables with the original cytokine levels.

• *To identify individuals with similar profiles of immunological parameters *and to place them into groups (so called "clusters"). For example, patients with a clinical outcome might be defined as "atopics" or "non-atopics" based on the values of skin prick tests; or subjects with a specific infection may be classified into groups (eg, active, chronic, or past) defined by the overall elevation in antibodies (e.g. IgE, IgA, IgM or IgG subclasses). However, within the same group of patients, clusters with distinct or overlapping profiles might be distinguishable and subsequent analyses might show associations between distinct clusters and disease (or some other outcome).

ii) The second group of research objectives *investigates causal relationships *between one or more immunological parameters (e.g. different cytokines, or summary measures) and other study variables (e.g. an outcome such as asthma). To guide the investigation of causation it is important to have developed an *a priori *causal pathway model. This will allow the appropriate definition of variables, i.e. defining which variables are dependent variables (outcomes), intervening variables (mediating the effect) or independent variables (exposures, confounding factors and effect modifiers) and will determine the choice of statistical approach.

Possible research objectives for causality include:

• *Identification of determinants of immunological profiles*. The objective may be to compare the expression of cytokines between two or more groups defined by an exposure, e.g. people infected or not infected with helminths, or people vaccinated and non-vaccinated in a vaccine trial where the vaccine exposure is assumed to influence the levels of the immunological parameter to be measured. For example, the question may be to determine if BCG vaccination influences the levels of IFN-*γ *secreted by mononuclear cells stimulated in vitro with a mycobacterial antigen. The immunological parameter is *the outcome *or *dependent variable*.

• *Identification of clinical consequences of immunological profiles*, (immunological parameter as the risk factor) or, in other words to identify associations between an immunological parameter and clinical (or other) outcomes. For example, immunologists are interested in predicting the probability of a disease occurrence by measurement of cytokine levels. For example, whether elevated TNF-*α *levels are associated with active disease in rheumatoid arthritis? The immunological parameter is *the risk factor (often called "exposure") *or *independent variable*.

iii) The third group consists of *more complex research questions *that may include two or more of the objectives described above. Such questions may examine the role of cytokines in larger causal constructs, including more than one risk factor, intervening variables that mediate and modify an effect, and outcomes; and inter relationships between them. An example will be to investigate the causal inter relationships between early life infections, level of expression of pattern recognition receptors (such as Nods), activity of pro-inflammatory cytokines (IFN-*γ *and TNF-*α*) and the development of inflammatory bowel disease.

iv) The field of *in silico *immunology (computer analysis generally in conjunction with informatics or immuno-informatics) is a rapidly developing and expanding field and has been used to address several types of study questions, such as:

• The prediction of immunogenic sequences from microbial genomes to predict potential vaccine candidates [9].

• The prediction of protein sequences in therapeutic antibodies that may be associated with adverse reactions [10].

• Identification of regulatory molecules in the innate immune system [11].

These approaches are generally high-throughput analyses of large data sets (e.g. microbial genomes, human genome, etc) using available software (e.g. EpiMatrix) to either generate or test hypotheses and have been reviewed in detail elsewhere [12-15].

*In silico *statistical analyses use many of the multivariate statistical techniques discussed later in this review (e.g. cluster analysis). Because this is a highly specialised field for which there are many computational tools available [9], *in silico *immunology will not be discussed further in this review.

## Statistical methods for analysis of immunological data

We conducted a systematic literature search in the database MEDLINE (1980–2005) to review statistical methods that have been previously applied to cytokine data. Because the objective was to get a crude overview rather than to reveal the exact number of papers published in this area we defined quite sensitive search criteria using the following key words: *"cytokine$" *or terms to identify specific cytokines (e.g. among others *"IL$," "interleukin$," IF$, interferon$, TNF$*, etc.) and common univariate and multivariate statistical techniques (e.g. among others *"linear regression,""analysis of variance,""cluster analysis,""factor analysis" *etc.).

Table 1 shows the results of our search. The most widely used methods found were simple statistical approaches that investigate the relationship between two variables (so called *bivariate meth*ods – also called *univariate methods *when variables are classified as dependent and independent variables). We frequently found standard methods to compare means of immunological parameters between independent groups (e.g. t-test, analysis of variance or their non-parametric equivalents), bivariate correlation analysis (Pearson's or Spearman's correlation coefficients) and univariate linear regression. By contrast, *multivariate techniques *( i.e. statistical approaches that consider three or more study variables simultaneously) were less frequently applied to cytokine data. Several studies used factor analysis (to identify groups of correlated immunological parameters) or cluster analysis (to identify groups of individuals with similar immunological profiles) or discrimination techniques such as logistic regression, discriminant analysis (to identify causes or consequences of immunological profiles). We also found a few examples of advanced modelling techniques (path analysis/structural equation modelling) that simultaneously model multiple relationships between the study variables.

**Table 1:** Results of a literature review conducted in the medical database MEDLINE (1980–2006) about statistical methods found in immunological studies investigating cytokine expressions.

***Statistical methods***	**Number of references**
**Univariate techniques**	
	
Analysis of variance	2908
T-test	420
Mann-Whitney U-test	316
Wilcoxon/McNemar test	193
Univariate linear regression	163
Bivariate correlation analysis	157
Kruskal-Wallis H-test	95
Repeated measures analysis of variance	31
Friedman test	7
Non linear regression	5
	
**Multivariate techniques**	
Logistic regression	629
Cluster analysis	192
Multivariate analysis of variance	144
Multiple linear regression	91
Factor analysis/Principal components analysis	80
Analysis of covariance	56
Linear discriminant analysis	51
Partial correlation coefficient	24
Multinomial logistic regression	9
Multivariate analysis of covariance	7
Path analysis/Structural equation modelling	4

In the following section we provide an overview of statistical methods that can be considered for analysing immunological data that should help the applied immunologists without a detailed knowledge of statistics to select the appropriate statistical technique for each particular research question. The definition of which method is the most appropriate is strongly dependent on the research objective, the type of data collected, whether data assumptions are fulfilled and whether the sample size is sufficient. We begin with a short introduction to these topics.

### Exploratory Data Analysis

An important first step in analysing immunological data is exploring and describing the data. Whatever the research question investigators should first explore the data in tables showing summary statistics (e.g. means, standard deviations, etc.) and apply graphical methods such as bar charts, histograms, Box and Whisker plots, or scatter plots. For multivariate data, scatter plot matrices are a powerful tool to examine associations among several immunological parameters [16]. A good overview of statistical methods for Exploratory Data Analysis is provided by Tukey [17].

### Data Assumptions

The next thing investigators have to consider when selecting a statistical method is whether their data meet a number of *data assumptions*. The first assumption is the *scale of measurement *of the data, i.e. whether *the data type *is categorical (e.g. groups like male and female), ordinal (e.g. groups with a logical order, like order of birth) or continuous (also called metric [measured on a defined scale]). This restricts the statistical methods that can be used. When the investigator has to deal with continuous data the second assumption to test is whether the data follow a theoretical distribution (e.g. normal distribution). Distributional assumptions can be tested graphically by diagnostic plots or by applying a statistical test that compares the distribution of the data with a theoretical distribution. When the original data do not meet distributional assumptions required for a particular statistical technique (e.g. normal distribution to apply a t-test) a common approach is transforming the data to meet the data assumptions, for example to use the logarithm of the values [4]. However, immunological data frequently do not meet data assumptions even after applying different data transformations. In such cases it may be more appropriate to use an alternative statistical approach that requires fewer data assumptions (e.g. applying a non parametric Wilcoxon test instead of a t-test) [5]. Another approach for continuous variables that do not fulfil distributional assumptions is "categorising" the measurements by biological meaningful cut-off values (e.g. level >= 2.5: "positive", level < 2.5: "negative") or using centiles, and then apply a statistical test that is appropriate for categorical data. A less frequently applied strategy that allows for the use of a parametric approach even when data assumptions are violated is the "robust resampling variance estimator [18]." The concept behind this approach is to draw repeated random samples from the data, and then to estimate the parameter of interest (e.g. the mean) from each sample and finally to obtain an estimate of the variance by calculating the variance of the parameter across the samples. "Robust estimation" means the approach will provide valid estimations even when data assumptions (e.g. normal distribution) are violated [3]. For example, in a hypothetical immunological study IL-10 measurements have been obtained from a sample of 100 individuals. By drawing 500 random samples from these 100 individuals each including 50 subjects, a robust estimate of the variance of the parameter of interest (the population mean) can be calculated from the mean of each sample and from the variance of the mean (across the samples).

### Sample size issues

A second major issue that immunologist frequently face and that also substantially affects the statistical approach to be used is to estimate the appropriate sample size for a statistical analysis. Whereas for univariate techniques (e.g. t-test, ANOVA etc.) sample size can be determined by conducting a power analysis [19], sample size formulae are available only for a couple of multivariate techniques. *Cohen's Power analysis guide *is a useful review for calculating sample sizes for common univariate and some multivariate techniques (analysis of covariance and multiple regression) [20]. Unfortunately, the theory underlying sample size estimation for other multivariate techniques is less developed. Recommendations for sample size for studies using factor analysis are that more subjects should be included than the number of unique correlations present in the correlation matrix [21,22]. Authors suggest that for cluster analysis sample size calculations are dependent on how the investigator believes the study population is clustered. If some small clusters (say less than 10) are expected, then sufficient subjects will be required to sample at least 5 to 10 people in the smallest cluster [23]. There is a statistical framework and software available for sample size calculation for advanced techniques such as structural equation modelling and path analysis [24,25].

## Selecting the appropriate statistical method for each particular research question

**Figure 1:**
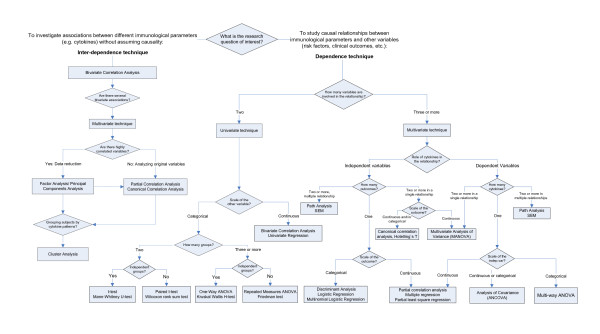
Selecting the appropriate statistical technique for analysis of immunological data.

In this section we provide an overview of statistical methods that we consider useful for statistical analysis of immunological data. Our guide for data analysis was written with the "classical statistical approach" in mind, i.e. we provide statistical techniques to extract the maximum information from the present data. We do not discuss *Bayesian statistical methods *that also might be useful for application to immunological data seeking to combine *a priori *information with the information captured in the present data; a good overview is provided by Lee [26]. Moreover, statistical methods are important also for standardising immunological techniques and this is discussed elsewhere [27]. We have prepared a summary table (Table 2) that lists the most important univariate and multivariate methods and links them to typical immunological research questions. In addition, we provide a flowchart that will help the immunologist to select the appropriate statistical analysis according to the research objective and the number and type of study variables to be analysed (Figure 1). Most examples refer to cytokine data but the application to other continuous immunological data is straightforward.

**Table 2:** Selection of important statistical methods suitable for the analysis of immunological data.

**Example of research question**	**Type of data **[D: dependent, I: independent]	**Other data assumptions**	**Statistical method^1^**
**Univariate techniques**			
*Univariate group mean comparison techniques*			
Compare expression of a cytokine between two independent groups (e.g. treatment vs. control)	D: continuous I: categorical	Normal distribution homogeneity of variances	t-test
	D: continuous or ordinal I: categorical		Mann Whitney-U test
Compare expression of a cytokine between two related groups (e.g. before and after treatment)	D: continuous I: categorical	Normal distribution, homogeneity of variances	Paired t-test
	D: continuous or ordinal I: categorical		Wilcoxon rank sum test
Compare expression of a cytokine between three or more independent groups defined by one factor (e.g. treatments A, B, C)	D: continuous I: categorical	Normal distribution, homogeneity of variances	One-way analysis of variance
	D: continuous or ordinal I: categorical		Kruskal Wallis – H test
Compare expression of a cytokine between three or more related groups (e.g. measurements 1, 2, and 3 weeks after treatment)	D: continuous I: categorical	Multivariate normal distribution, assumptions about covariance	Repeated measurements analysis of variance
	D: continuous or ordinal I: categorical		Friedman's ANOVA
*Correlation and regression analysis*			
Quantify association between two cytokines or a cytokine and another continuous variable	D: continuous I: continuous	Linear relationship, normality	Pearson correlation coefficient
	D: continuous or ordinal I: continuous or ordinal	Linear relationship	Spearman rank correlation coefficient
Predicting expression of a cytokine by a continuous independent variable	D: continuous I: continuous	Specified relationship (e.g. linearity for linear regression), normal distribution (for parametric regression)	Univariate regression
**Multivariate techniques**			
*Multivariate correlation and regression techniques*			
Quantify associations between two cytokines adjusted for the effect of a third continuous variable	All variables: continuous	Linear relationship, normality	Partial correlation coefficient
Predicting a continuous outcome (e.g. a cytokine) by several continuous or categorical independent variables	D: continuous I: continuous, ordinal or categorical	Specified relationship (e.g. linearity for linear regression), normal distribution for parametric regression, No multi-collinearity	Multiple regression
		Specified relationship, multi-collinearity	Partial least squares regression
Quantifying the magnitude of correlation between two groups of continuous variables (e.g. Th1 and Th2 related cytokines)	All variables: continuous		Canonical correlation analysis
*Multivariate group mean comparison procedures*			
Compare cytokine expressions between three or more independent groups defined by two or more factors (e.g. treatment and gender)	D: continuous I: categorical	Normal distribution, homogeneity of variances	Multi-way analysis of variance (ANOVA)
Simultaneously compare expressions of two or more cytokines between three or more independent groups defined by two or more factors	D: continuous I: categorical	Multivariate normal distribution, homogeneity of covariance matrices	Multivariate analysis of variance (MANOVA)
Compare cytokine expressions between three or more related groups defined by two or more factors (e.g. measurements at different time points during a study and treatment)	D: continuous I: categorical	Multivariate normality, homogeneity of covariance matrices	Multi-way repeated measurements analysis of variance
Grouping set of correlated cytokines to summary variables ("principal components")	All variables: continuous	High degree of multicollinearity	Factor analysis/Principal components analysis
Grouping subjects in homogenous subgroups according to similar expression levels of two or more cytokines	All variables: continuous	Low degree of multicollinearity	Cluster analysis
*Classification procedures*			
Explaining or predicting group membership of two or more independent groups by cytokine levels	D: categorical I: continuous	Multivariate normal distribution, equal covariance matrices, low degree of multicollinearity	Linear discriminant analysis
Explaining or predicting group membership of two independent groups by cytokine levels	D: categorical I: continuous, ordinal or categorical		Logistic regression
Explaining or predicting group membership of three or more groups by cytokine levels	D: categorical I: continuous, ordinal or categorical		Multinomial logistic regression
*Advanced techniques for multiple relationships*			
Modelling multiple relationships between several immunological parameters and one or more outcome variables	All variables: categorical, ordinal or continuous data	Conceptual framework specifying the multiple relationships among the study variables	Path analysis/Structural equation modelling

^**1**^All univariate and multivariate statistical approaches listed above can be implemented in general purpose statistical packages, e.g. among others *S-PLUS*^® ^(Insightful Corporation, Seattle, WA), *SAS*^® ^(SAS Institute Cary, NC, USA), *SPSS*^® ^(Chicago: SPSS Inc.) or *STATA*^® ^(StataCorp. *Stata Statistical Software*. College Station, TX: StataCorp LP). Path analysis/structural equation modelling can be implemented in *STATA *and *SPSS *that provide the extensions modules *GLLAMM *and *AMOS*, respectively, as well as in several special purpose software packages, e.g. among others *LISREL*^® ^(Scientific Software International, Inc, IL, USA) or MX^® ^(MCV, Department of Psychiatry, Richmond, VA, USA).

### Inter-dependence techniques

The first group embraces so called *inter-dependence techniques*, i.e. statistical methods aimed to explore relationships between study variables without assuming any causal relationship. These techniques are appropriate when the investigator cannot define (or may not wish to define) which variable is the independent variable (cause, exposure) and which is the dependent variable (effect, outcome). For example, inter-dependence techniques might be very useful in immunological studies to examine relationships between different cytokines measured in the same individual.

#### Correlation analysis

One commonly applied inter-dependence approach is *bivariate correlation analysis *that aims to assess the magnitude of the linear relationship between two continuous variables (e.g. two cytokines, or a cytokine and another continuous variable. For example, Hartel *et al *conducted an observational, cross-sectional study in children aged between 1 and 96 months and in adults to investigate age-related changes in cytokine production [2]. The association between cytokine levels and age was analyzed using non-parametric rank correlation coefficients.

If there are more than two immunological parameters of interest and one finds several significant bivariate correlations, a *multivariate correlation analysis *should be conducted to examine the degree of multicollinearity in the data, i.e. whether multiple relationships are present between three or more study variables. A simple but useful approach to examine associations between three variables is to conduct stratified bivariate correlation analysis across strata defined by levels of a third variable. For example, to examine the association between a Th1-related and a Th2-related cytokine could be examined after stratifying by low and high levels of expression of IL-10.

#### Data reduction techniques

Datasets with several highly correlated immunological parameters can be simplified using "*data reduction techniques."* These methods are especially appropriate when it is assumed that many variables reflect aspects of an underlying process which is not directly measured. The most common technique is *Principal Component Analysis (PCA) *[28], which is a special type of *Factor Analysis *[29]. The idea behind this approach is to create summary variables (called "principal components"), that capture most of the information of the original data. For example, the technique can be used to derive two principal components from several correlated cytokines. After using this technique it is essential to consider whether the components identified are biologically plausible (e.g. it would be important to observe that the classification of cytokines grouped into two groups is consistent with the findings of published literature).

A useful feature of factor analysis and especially PCA is that the weights (the "factor loadings") for each variable within the components can be interpreted as correlation measures between the observed variable and the underlying unobservable component. Data assumptions in factor analysis are more conceptual than statistical. From a statistical point of view, normality is only necessary if a statistical test is applied to measure the significance of the factors, but these tests are rarely used in practice. The more important conceptual assumptions are that some underlying structure does exist in the set of selected variables and that results in some degree of multicollinearity.

In immunological studies factor analysis or PCA could be applied to extract information on the Th1- or Th2-related immune response from a set of cytokines or the ratio of a Th1- and Th2-score could be calculated to quantify the degree of "Th1/Th2 bias [30,31]." For example, Turner *et al *[31] derived summary variables (called "principal components") from 11 different cytokines that were believed to better reflect the underlying mechanism and that were used in further analyses [32] instead of the original parameters. Data reduction techniques should be used when the objective of the study is not to investigate the role of each parameter but the role of the underlying mechanism.

#### Cluster Analysis

*Cluster Analysis (CA) *is the appropriate statistical approach when the researcher seeks to group individuals (not variables) according to their values of study variables (e.g. cytokine levels) [23]. CA groups individuals so that subjects in the same cluster have similar profiles of the parameters being studied (i.e. a high "within-cluster homogeneity") and subjects from different clusters have quite different immunological profiles (i.e. a high "between-cluster heterogeneity"). To perform CA the researcher has to define the variables on which the clustering is to be based and the type of cluster algorithm. *Agglomerative algorithms *treat each observation as a cluster and group similar individuals into clusters, while *divisive algorithms *start with the whole study population as a single cluster and divide the population by identifying homogeneous subgroups. The most appropriate clustering approach for a particular dataset depends on the type of data collected and the research question. Agglomerative clustering is preferable when there are extreme values in the data (outliers).

CA has strong mathematical properties but not statistical foundations. Data assumptions (e.g. normality and linearity) that are important in other multivariate techniques are of little importance in cluster analysis. However, the researcher is encouraged to examine the degree of multicollinearity in the data because each variable is weighted and variables that are multi-collinear are implicitly more heavily weighted in the clustering algorithm. For example, a cluster solution derived from a dataset with five highly correlated Th1-related cytokines and two correlated Th2-cytokines would substantially overestimate the importance of the Th1-component in the clustering. This can be avoided by first applying a data reduction technique (e.g. PCA) to derive the "principal components" that quantify the magnitude of Th1/Th2-immune response and afterwards clustering the individuals with respect to these immunological components.

In immunological studies cluster analysis may be useful to identify groups of individuals with similar immunological patterns (e.g. cytokine or antibody levels) that reflect an unknown common underlying immunological mechanism. For example, Mutapi *et al *[16] sought to group people infected with *S. mansoni *into clusters defined by levels of parasite specific IgE, IgA, IgM and the IgG subclasses. The authors identified two clusters, a cluster with high levels of IgM and low levels of all other antibodies and a cluster with high IgM and IgG1 and medium IgG4 and low levels of all other antibodies. In further analyses the authors investigated whether epidemiological features of schistosomiasis were associated with cluster membership and whether treatment changed this.

Another useful technique to study associations among immunological parameters without assuming any causal relationship is *Canonical Correlation Analysis (CCA) *[33] an approach that aims to quantify the correlation between two predefined sets of variables. In our MEDLINE search we did not find any previous applications of CCA to immunological data but we suggest that this statistical approach might be very useful to quantify the magnitude of correlation between two different sets of immunological parameters. A hypothetical example is where the investigators were interested to quantify the correlation between Th1- and Th2-related cytokines in individuals with and without helminths infections, or in atopic or non-atopic study subjects.

### Dependence techniques

The second group of techniques are statistical *dependence techniques *that are appropriate when the study investigates causation, i.e. variables can be classified as independent (cause, exposure) and dependent variables (effect, outcome), based on concrete *a priori *hypotheses about the underlying biological mechanisms. For example, an immunological parameter might be considered as an independent variable when it is the proposed cause (e.g. an autoantibody in an autoimmune disease) or the dependent variable when it is considered to be an effect (or outcome [e.g. the production of interferon gamma produced by lymphocytes stimulated with mycobacterial antigen following BCG vaccination]), or an intervening variable or intermediate factor (mediating variable) within a complex causal chain (e.g. the cytokine IL-13 would be an intermediate factor in studies that examine the relationship between exposure to aeroallergens and the development of atopic asthma). For example, Black *et al *[34] studied the IFN-*γ *response to *Mycobacterium tuberculosis *(as the outcome variable) before and after receiving BCG vaccination (the independent variable). Cooper *et al *[35] studied the effect of cholera vaccine (the independent variable) on the IL-2 response to recombinant cholera toxin B (as the outcome). In both studies, the authors pre-specified the classification of study variables before conducting statistical analysis. A more complex hypothetical example is the investigation of the effect of the impact of the intensity of infection with helminths on cytokine expressions (e.g. using Th2-related cytokines as outcomes) in a study of the relationship between helminths infections and atopy, and when the investigators may wish to consider the same cytokines as determinants, or risk factors, for atopy within the same study.

The choice of which statistical dependence technique is most appropriate for a particular research question will depend on the study design, the number and "scaling" (i.e. continuous, ordinal or categorical) of the study variables and other data assumptions (see Figure 1).

### Univariate dependence techniques

These methods are appropriate when there is only *one dependent *and *one independent *variable. Common techniques are *univariate group mean comparison procedures *[36] (aimed to compare the levels of a continuous variable (e.g. cytokine expressions) between groups of individuals pre-defined by an exposure that is considered to cause the immunological profile (e.g. vaccinated or not). The number of groups and whether the groups are independent or related will determine which approach is the most appropriate for each situation (see overview in Table 2).

By contrast, *univariate regression analysis *[37] is the best approach when the investigator seeks to model the relationship between two continuous variables and is able to decide which one is the outcome variable. The most common approach is *Linear Regression *a technique with stringent data assumptions (linearity of the relationship and normality of the error distribution). Robust alternatives when data assumptions are violated are non-parametric or non-linear regression techniques. Examples of applications for regression analysis in immunological studies are predicting the levels of expression of a continuous outcome variable (e.g. a cytokine) by a continuous variable (e.g. age or another continuous immunological parameter) when a causal relationship can be assumed.

***Multivariate dependence techniques ***are needed when there are three or more variables involved and at least one variable can be considered the dependent variable.

#### Classification techniques

These methods are required when there are several independent variables (e.g. cytokines) and one categorical outcome (e.g. atopy). One classical approach is *Linear Discriminant Analysis (LDA)*, a method that derives linear combinations of the independent variables (called discriminant functions) that best discriminate between the two outcome groups (defined on the basis of the independent variable) [38]. LDA requires continuous normally distributed independent variables. A flexible approach which can be used with non normally distributed data and involves categorical independent variables is *Logistic Regression *[39].

In immunological studies classification techniques can be very useful to identify immunological profiles that best discriminate two or more pre-defined groups of interest (e.g. atopy vs. non atopy). For example, Gama et al [40] used LDA aimed to identify an immunological marker based on six cytokines (IL-2, IL-4, IL-10, IL-12, IFN-*γ *and TNF-*α*) to discriminate between clinical and asymptomatic forms of visceral leishmaniasis. The authors found that TNF-*α*, IL-10 and IL-4 were highly correlated with the clinical form, while IL-2, IL-12 and IFN-*γ *were correlated with the asymptomatic form. In another study, logistic regression was used to explore the role of parasite induced IL10 in decreasing the frequency of atopy: Van Biggelaar *et al *[41] sought to predict positive skin prick tests to house dust mite in children by mite-specific IgE, total IgE, IL-5 and IL-10 to worm and used logistic regression to show that positive skin prick test was positively associated with mite specific IgE but negatively associated with IL-10; and the probability of a skin test positivity was a result of the interaction between level of mite IgE and worm IL10.

#### Multivariate group mean comparison techniques

These techniques such as *Multivariate Analysis of Variance (MANOVA) or Multi-way Analysis of Variance (Multi-way ANOVA)*, are used to compare the distributions of one or more continuous variables between groups defined by one ("one-way") or more ("multi-way") factors of interest (see overview Table 2) [21]. In contrast to LDA, where the groups are assumed to define a categorical outcome (e.g. asthmatic vs. non asthmatic) and the independent variables (e.g. cytokines) are used to discriminate between groups, in (M)ANOVA the groups are defined by the investigator considering one or more independent variables (e.g. treatments, vaccination status). A very useful application of MANOVA in immunological studies is to simultaneously compare the levels of two or more cytokines (e.g. IFN-*γ*, TNF-*α*) between two groups (asthmatic vs. non asthmatic). By applying MANOVA instead of repeated application of ANOVA the investigator can avoid the problem of type I error inflation for the whole experiment.

*Multivariate Analysis of Covariance *(MANCOVA) is an extension of MANOVA that additionally allows to control for the effect of an other continuous variable to be controlled (e.g. a confounder) [21]. An application of MANCOVA in immunological studies could be to simultaneously compare the expression levels of different cytokines (e.g. IF-*γ*, TNF-*α*, etc.) across groups defined by one or more experimental factors (e.g. vaccinated or control) and adjusted for age.

#### Multiple Regression Techniques

*Multiple Regression *is appropriate when the research question is to predict a single continuous dependent variable by a set of continuous and/or categorical independent variables [37]. The standard approach frequently used is multiple linear regression, however there are alternatives (e.g. non-parametric, non-linear multiple regression) when data assumptions are not met [42]. Regression analysis could be applied in immunological studies to predict the expression of a cytokine by explanatory variables (e.g. a set of other cytokines or other immunological parameters) or to predict the values of a continuous outcome variable (e.g. intensity of parasitic infection) by the expressions of one or more cytokines. For example, Dodoo *et al *[43] used multiple linear regression to predict malaria-related outcomes (fever, hemoglobin concentration) by levels of different cytokines (IFN-*γ*, TNF-*α*, IL-12, IL-10, TGF-*β*).

*Partial Least Squares (PLS) Regression *[44] is an extension of multiple linear regression for constructing predictive models when the factors are many and highly collinear. The approach could be very useful for analysis of immunological data, e.g. when the objective is to predict an outcome by a large set of highly correlated immunological parameters. Technically, the approach is a combination between principal components analysis and multiple linear regression, i.e. it produces factor scores as linear combinations of the original predictor variables so that there is no correlation between the factor score variables used in the predictive regression model.

### Advanced techniques

#### Path analysis and Structural Equation Modelling

**Figure 2:**
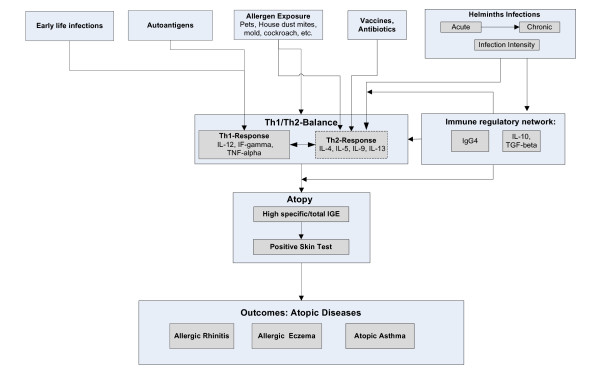
Conceptual framework that specifies multiple associations between potential risk factors, immunological parameters and outcomes (atopy and asthma).

All the multivariate statistical methods that have been mentioned above have one common limitation: although they may include many variables, they all assume the presence of one single relationship between them. However, in modern immunological studies investigators often assume *multiple relationships *among immunological parameters and other study variables so that a simple multivariate approach might not be sufficient to reflect the complexity of the underlying immunological process. For example, in an immuno-epidemiological study conducted to study risk factors for asthma and allergy, investigators could define a *conceptual framework *that assumes multiple relationships between risk factors (e.g. allergens, vaccines, early life infections), immunological profiles (e.g. cytokine expression levels) and the occurrence of outcomes (e.g. asthma, atopy) (Fig 2). To model such complex immunological processes it will be important to simultaneously model all these multiple associations. *Path Analysis *and *Structural Equation Modelling *(SEM) are techniques developed by geneticists [45,46] and economists [47] that can handle multiple relationships among study variables simultaneously and have been frequently applied in other scientific fields (e.g. economics, social sciences) [48]. SEM is an extension of path analysis that allows also for so called *"latent variables" *(a conceptual term for unobserved variables, see Appendix). A structural equation model consists of two components: "a measurement model" that defines how the observed measurements (called indicators) are related to the unobserved latent variables and a "structural model" that defines the assumed relationships between the observed study variables and one or more latent variables.

**Figure 3:**
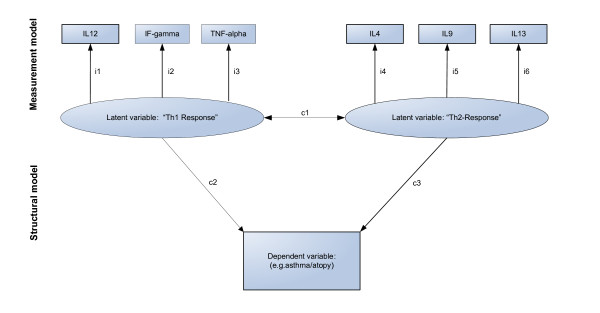
Example path diagram that specifies a structural equation model with two latent variables.

The concept of latent variables and SEM is likely to be useful in immunological studies because immunologists frequently hypothesise that the measured immunological parameters are the result of unobservable underlying complex immunological processes. For example, imagine a hypothetical immunological study where different cytokines are measured to quantify two important immunological components. Figure 3 shows the *path diagram *of the study in which multiple relationships are assumed between the study variables. In this example we consider that the cytokines IL-12, IFN-*γ *and TNF-*α *represent an unobservable latent variable "Th1-related immune response" and the cytokines IL-4, IL-9 and IL-13 represent the latent variable "Th2-related immune response". Further, the structural model assumes relationships between the two latent variables (c1) and the "effects" (c2, c3) of these on an outcome variable (e.g. atopy, asthma). In SEM the concept of the "measurement model" is similar to factor analysis in which a linear relationship between the (observed) indicator variables and the (unobserved) latent variable is assumed. For example, in Figure 3, the "indicator loadings i1–i6" reflect the magnitude of association of each cytokine with the latent variable. However, the distinction between the two analyses is that in factor analysis the principal components are extracted to maximize the degree of variance explained by a specified number of factors while in SEM the investigator has to define *a priory *a path diagram that specifies which variables are the indicators of the underlying latent variables and the correlations of the measurements with the latent variables are derived that best reflect the whole conceptual framework e.g. maximizing the correlation between all latent and observed variables.

Application of these techniques to immunological data should allow the inference of complex immunological phenomena. For example, Chan *et al *[49] used SEM to study the role of obesity-associated dyslipidaemia, endothelial activation and two cytokines (IL-6 and TNF-*α*) in a complex causal chain of the metabolic syndrome. There are few applications of conceptual frameworks, path analysis and SEM to immunological data in the literature. However, because of increasing sample size and advancing knowledge about underlying complex immunological mechanisms, these approaches have a potentially important role in the analysis of data from modern immunological studies. The concept of latent variables in SEM will be especially useful because immunological mechanism can rarely be observed directly and must therefore be inferred through the measurement of different immunological indicators. Moreover, the unique feature of SEM that allows the analysis of multiple relationships between the study variables would be appropriate for the inference of causal chains of immunological processes.

#### Mixed Effects Models

The application of *Random or Mixed Effects Models *[50] for statistical analysis of clustered or longitudinal data is becoming a popular method in medicine. The basic idea of the approach is to adjust data analysis for an effect of the clustering by introducing a "random effect", i.e. an unobserved random variable that is specific to the clustering unit. In immunological studies the application of mixed effects models could be very useful when clustering must be assumed in the data, e.g. measurements of different cytokines from the same patients, repeated measurements of the same cytokine in a longitudinal study or in multi-centre studies where patients have been recruited from different populations.

## Conclusion

The aim of this paper is to provide a modern overview for applied immunologists to explain and illustrate the statistical methods that can be employed for the analysis of immunological data. Our review should help immunologists without a detailed knowledge of statistics that are faced with the problem of statistical analysis of immunological data to select the appropriate statistical technique that will allow the valid extraction of the maximum information from the data collected. However, the statistical framework presented here should not be used as a substitute for an experienced biostatistician who should be involved from the beginning of the study for advice on study design, calculation of the sample size and planning of the statistical analysis. The systematic literature review illustrates the fact that most immunological studies still employ simple statistical approaches to immunological data even when multiple inter-relationships among several study variables are expected. We think it is important that more sophisticated statistical techniques are used for complex immunological data that will permit a better understanding of complex underlying immunological mechanism. Our focus has been on multivariate techniques that permit the analysis of multiple study variables simultaneously and hope that our examples from both real and hypothetical immunological studies will stimulate immunologists to make more use of these techniques. Moreover, bearing in mind the complexity of research questions addressed by modern immunological studies, we have introduced the idea of conceptual frameworks, latent variables and advanced statistical techniques (e.g. path analysis, SEM), providing a toolbox that should help the investigator to analyse multiple relationships among several study variables simultaneously. Finally, it should be pointed out that statistical techniques are a tool for the inference of underlying mechanisms and can never substitute for *a priori *hypotheses that are based on a sound knowledge of the scientific literature.

## Authors' contributions

BG and LCR had the idea for the paper, conducted the literature review, developed the guide for statistical analysis and wrote the manuscript. PJC and MY participated in writing the sections about research objectives of immunological studies and specific aspects of immunological data and evaluated the applicability of the proposed statistical framework for data from modern immunological studies. MLB participated in developing the statistical framework and helped to draft the manuscript. All authors have read and approved the final manuscript.

## Supplementary Material

Additional file 1 Appendix: Glossary of statistical and epidemiological terms. Click here for file
