# Supplementary material for: A guide to modern statistical analysis of immunological data
Source: BMC Immunol. 2007 Oct 26;8:27. doi: 10.1186/1471-2172-8-27 (PMC2234437; doi:10.1186/1471-2172-8-27)
Supplement: Additional file 1 — Appendix: Glossary of statistical and epidemiological terms. [file 1471-2172-8-27-S1.doc]

Appendix: Glossary of statistical and epidemiological terms.

| **Term** | **Explanation** |
| --- | --- |
| Dependent/Independent  variable | In [research](http://en.wikipedia.org/wiki/Design_of_experiments) one or more dependent variables (also called outcomes, response variables) are usually assumed to be affected by one or more independent variables (covariables, explanatory variables). In statistical analysis, investigators try to explain how the values of independent variables influence the values of the dependent variables. |
| Confounder | A variable in a statistical or research model that is not the main outcome nor the main explanatory variable but that affects the main variables of interest. Confounders that are not acknowledged and included in the analysis (“controlled for”) distort the results causing that the two measured variables falsely appear to be in a [causal](http://en.wikipedia.org/wiki/Causality) relation. |
| Latent variables | As opposed to [observable variables](http://en.wikipedia.org/wiki/Observable_variable), [variables](http://en.wikipedia.org/wiki/Variable) that are not directly observed but are inferred from other variables that are observed and directly measured. |
| Exposure | Variables describing the condition of being exposed (e.g. to be at risk of acquiring a disease). For example “exposure” to smoking increases the risk of lung cancer. |
| Risk factor | A risk factor is a variable associated with an increased risk of [disease](http://en.wikipedia.org/wiki/Disease), [infection](http://en.wikipedia.org/wiki/Infection), death, etc. Risk factors are not necessarily causal, for example, being young cannot be said to cause measles, but young people are more at risk. |
| Variance estimate | Measure for the variability of a random variable, estimated in a population of subjects by calculating the mean of the squared deviances from the mean. |
| Conceptual framework | Hierarchical system of study variables (e.g. outcomes, risk factors, confounders, latent variables, etc.) and relationships between them defined to describe a complex phenomenon (e.g. an immunological process. |
| Path diagram | Graphical representation of a conceptual framework visualizing the relationships among the study variables. |
| Multicollinearity | Multiple correlations among different study variables |
